# Probing the Surface of Human Carbonic Anhydrase for Clues towards the Design of Isoform Specific Inhibitors

**DOI:** 10.1155/2015/453543

**Published:** 2015-02-24

**Authors:** Melissa A. Pinard, Brian Mahon, Robert McKenna

**Affiliations:** Department of Biochemistry and Molecular Biology, College of Medicine, University of Florida, P.O. Box 100245, Gainesville, FL 32610, USA

## Abstract

The alpha carbonic anhydrases (*α*-CAs) are a group of structurally related zinc metalloenzymes that catalyze the reversible hydration of CO_2_ to HCO_3_^−^. Humans have 15 different *α*-CAs with numerous physiological roles and expression patterns. Of these, 12 are catalytically active, and abnormal expression and activities are linked with various diseases, including glaucoma and cancer. Hence there is a need for CA isoform specific inhibitors to avoid off-target CA inhibition, but due to the high amino acid conservation of the active site and surrounding regions between each enzyme, this has proven difficult. However, residues towards the exit of the active site are variable and can be exploited to design isoform selective inhibitors. Here we discuss and characterize this region of “selective drug targetability” and how these observations can be utilized to develop isoform selective CA inhibitors.

## 1. Introduction

Carbonic anhydrases (CAs, EC 4.2.1.1) are a family of ubiquitous, mostly zinc metalloenzymes that catalyze the reversible hydration of carbon dioxide to bicarbonate and a proton [1, 2]. These enzymes are expressed in most living organisms and are encoded by five evolutionary distinct gene families: *α*-, *β*-, *γ*-, *δ*-, and *ζ*-CAs [3–5]. The *α*-CAs are expressed predominantly in vertebrates and are the only class observed in humans. *β*-CAs are found in prokaryotes, algae, and plants [6]; the *γ*-CAs are present in archaebacteria [7], while the *δ*- and *ζ*-CAs are found in diatoms [8]. The *α*-CAs have been extensively studied due to their role in human physiology and disease pathology [9]. Humans express 15 different isoforms, 12 of which are catalytically active and differ in their enzymatic efficiency. These isoforms also differ in cellular distribution and physiological function (Table 1). Specifically, there are eight cytosolic (CA I, II, III, VII, VIII, X, XI, and XIII), two mitochondrial (CA VA, and VB), one secreted (CA VI), three transmembrane (CA IX, XII and XIV), and one GPI-anchored (CA IV) isoforms of CA [10]. CA VIII, X and XI are noncatalytic due to the absence of one or more of the coordinating histidine residues and are termed CA related proteins (CA-RPs) [11].

**Figure 1:**
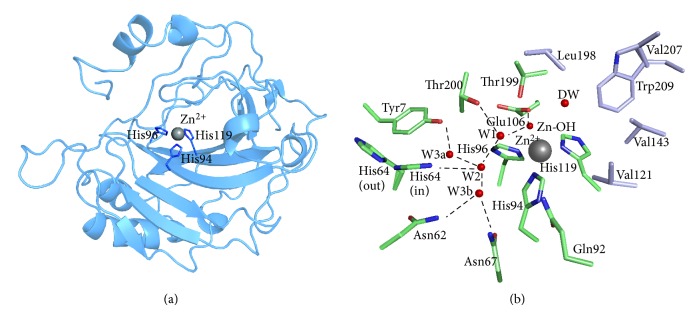
Structure of CA II. PDB ID: 3KS3. (a) Ribbon diagram depicting the overall structural fold. The active site zinc ion and coordinated histidines shown. (b) Active site and ordered waters (red spheres). Also shown are the hydrophilic (green) residues as well as the hydrophobic (purple) residues lining the active site.

The *α*-CA active site is located at the base of a large conical cavity spanning from the protein's surface to its center. This cavity is approximately 15 Å wide at its opening and 15 Å deep [4, 12, 13] based on observations in human CA II. At the core of the active site is a Zn(II) ion in a distorted tetrahedral coordination with His94, 96, and 119 (CA II numbering; used throughout) and a water/hydroxide molecule [14] (Figure 1). The active site of CA exhibits an amphiphilic nature and contains both a hydrophobic (Val121, Val143, Leu198, Val207, and Trp209 in purple, Figure 1(b)) and hydrophilic side (Tyr7, Asn62, His64, Asn67, Thr199, and Thr200 in green, Figure 1(b)) [15]. A high degree of residue conservation between the CA isoforms exists in each region.

**Figure 2:**
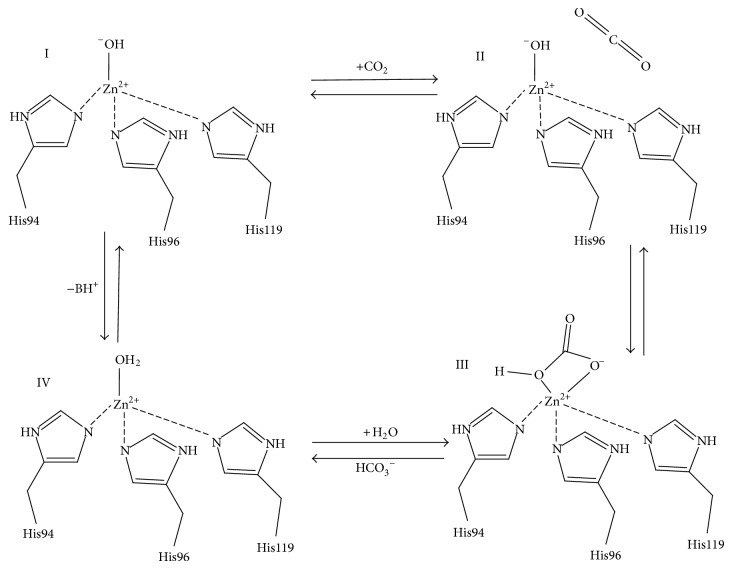
Schematic representation of CA catalytic mechanism.

The first step of catalysis by CA is the nucleophilic attack of a Zn-bound OH^−^ (active basic form) on a CO_2_ molecule, (Figure 2, I-II) to produce HCO_3_^−^ (III). The HCO_3_^−^ remains weakly bound to the Zn(II) ion (III) until it is displaced by a water molecule (III-IV) (inactive acidic form) and released into solution [16]. In the second step of CA catalysis (IV-I) the Zn-bound water regenerates to OH^−^ through a proton transfer event mediated by a highly conserved (in most isoforms). Histidine residue in combination with a network of ordered water molecules that are stabilized by the adjacent hydrophilic region of the enzyme's active site [1, 2, 15] (Figure 1(b)). In crystal structures of CA II, His64 has been observed to occupy two distinct positions: inward (pointing towards the active site) and outward (pointing away from the active site) conformations (Figure 1(b)). The general consensus is that the inward conformation of His64 is poised to accept the proton that has been transferred from the catalytic zinc to the water network, while the outward conformation is in an orientation that favors proton shuttling to the bulk solvent [16–18].

CAs are among the most efficient catalysts known, however there is variation in catalytic efficiency between isoforms such that the members of the *α*-CAs with the exception of the CA-RPs can be divided into three generalized categories. As such, CA II, IV, VB, and VII are among the fastest of the human CAs with CA II exhibiting a *k*_cat_ of 1.4 × 10^6^ sec^−1^. CA VA, VI, IX, and XII exhibit relatively intermediate catalytic activity, and CA III, XIII and XIV are considered the least efficient CAs [3, 9] (Table 1). The efficiency of these enzymes depends on the speed of proton shuttling during the two-step catalytic mechanism [3, 18]. In most of the CAs, this proton shuttling residue is the aforementioned histidine at position 64 [19–21]. In CA III, which is considered the slowest among the CA isoforms (<1% of CA II activity), a lysine is at position 64 [16].

**Figure 3:**
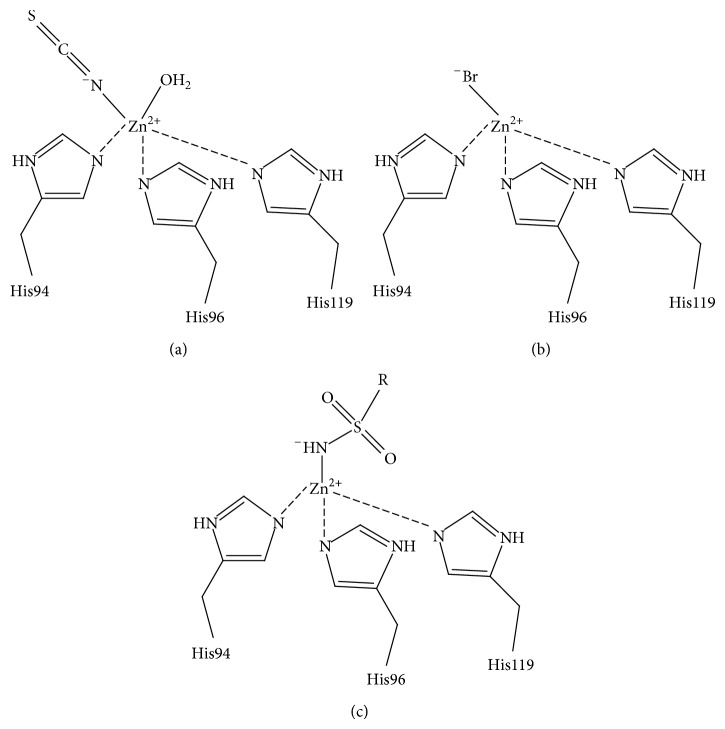
CA inhibition mechanism. (a) Anions such as thiocyanate form trigonal-bipyramidal adducts (b) Anions such as Br^−^ form distorted tetrahedral adducts (c) sulfonamides as well as some anions form regular tetrahedral adducts.

The human CAs are involved in various physiological functions, ranging from bone resorption to pH regulation, with abnormal levels or activities of these enzymes being commonly associated with various diseases (Table 1). Two main classes of CA inhibitors (CAIs) exist: the metal chelating anions and sulfonamide-based inhibitors. Both classes of CAIs are often referred to as “classical” inhibitors of CA and bind directly to the Zn(II) ion in the active site, displacing the Zn-bound solvent molecule. Metal chelating anions bind as either a trigonal-bipyramidal, distorted tetrahedral, or regular tetrahedral adduct [22] (Figures 3(a)–3(c)). Alternatively, sulfonamides generate a tetrahedral geometry upon binding to the catalytic zinc [9] (Figure 3(c)). This “classical” mode of binding of sulfonamide-based and anion CAI will be presented in more detail in later sections of this study.

As mentioned previously, the *α*-CAs display a remarkable diversity in regards to tissue distribution and overall physiological function. As such, a brief overview of each of these characteristics is presented and is summarized in Table 1.

Cytosolic CAs I and II are both expressed in red blood cells and are necessary for maintaining physiological pH of the blood through production of HCO_3_^−^ [23]. Abnormal levels of CA I in the blood are used as a marker for hemolytic anemia. CA II is ubiquitously expressed in other tissues including the kidney [24], bone, and also in ocular tissues [25]. Interestingly, CA II has also been shown to be associated with several transporters including the Cl^−^/HCO_3_^−^ exchanger, AEI [26], the Na^+^/HCO_3_^−^ cotransporter, NCB1 [27], and the Na^+^/H^+^ exchanger, NHE1 [28]. This suggests that CA II acts as a mediator of certain metabolic pathways by further providing the substrates for these various transporters [29]. As a result, CA II is often associated with several diseases such as glaucoma, renal tubular acidosis, and osteoporosis [3, 30]. In addition, CA II has also shown to be essential for the proper functioning of the water-transport channel, aquaporin-1 (AQP1) [31, 32]. Specifically, the relationship between CA II and AQP1 has been shown to be essential for maintaining proper CO_2_ transport in oocytes, regulation of AQP1 function, and also maintenance of a stable intracellular pH [31].

CA III expression is limited to skeletal muscle and adipose (both white and brown) tissue [33–35]. Unlike CA I and II, CA III displays (as mentioned previously) a remarkable 200-fold decrease in catalytic activity compared to CA II [36]. Furthermore, CA III contains two surface cysteine residues that can be glutathionylated thus acting as a vessel for reactive oxygen species (ROS) sequestration providing cell protection against oxidative damage [37]. These two attributes have caused speculation that CA III might serve a different physiological role unrelated to its primary catalytic function, although this notion is still unclear. It has been observed that CA III expression is directly correlated to adipogenesis and could potentially act as a regulator of peroxisome proliferator-activated receptor-*γ*2 (PPAR*γ*2) expression [38]. As a result CA III has not currently been linked to any particular disease. CA VII is primarily expressed in colon, liver, skeletal muscle, and in the brain [39]. CA VII exists as two forms; one form displaying the complete amino acid sequence and the other containing a 56 residue N-terminal truncation [39]. Like CA III, CA VII has two surface cysteines that can be glutathionylated suggesting that it too can act in preventing cellular oxidative damage [40]. Though the physiological role of CA VII remains unclear, evidence suggests that this enzyme plays a role in neuronal excitement through HCO_3_^−^ production [41]. HCO_3_^−^ can mediate electric current through channels that are coupled to gamma-aminobutyric acid (GABA_A_) receptors, and upon inhibition of CA VII interruption of the current-gated channel is induced causing a suppression of neural excitement [42]. As a result CA VII has been a proposed target for treatment of seizures and neuropathic pain [43].

CA XIII is another active cytosolic CA. CA XIII expression has been shown to be localized to the thymus, kidney, submandibular gland, small intestine, and predominantly in both male and female reproductive organs [44]. It has been postulated that CA XIII plays a significant role in pH regulation of reproductive processes including sperm mobility [45]. To date, no significant physiological function regarding CA XIII has been observed. However, it should be noted that downregulation of CA XIII has been seen in cases of colorectal cancer; however the significance of this observation has not yet been concluded [45].

The CA-RPs: CA isoforms VIII, X, and XI are also located in the cytosol. It has been observed that the CA-RPs are expressed predominantly in the brain and as mentioned previously show no catalytic activity. To date, no known physiological roles, or relation to particular disease have been established [11]. As a result, we will not focus on these isoforms.

CA VA and VB are the only isoforms expressed in the mitochondrial matrix of hepatocytes and adipocytes, respectively [46]. CA VA has been shown to be directly associated with ureagenesis such that it provides HCO_3_^−^ to be utilized by carbamoyl phosphate synthetase I [47, 48]. Carbamoyl phosphate synthetase is responsible for synthesis of carbamoyl phosphate which is the rate-determining step of ureagenesis [47]. Furthermore, it has been shown that other necessary carboxylase reactions, including that of pyruvate carboxylase for gluconeogenesis, could be mediated by CA VA activity [48]. This indicates that CA VA can act as a key mediator in several metabolic pathways of the liver. In addition the same effect is seen in the mitochondria of adipocytes where CA VB facilitates carboxylase activity and thus causes induction of lipogenesis [49]. The relationship of CA VA and VB with certain metabolic pathways suggests that both enzymes could be considered as drug targets for modulating both gluconeogenesis and lipogenesis in cases of obesity and insulin resistance [50].

CA VI is the only CA that is secreted and has been found in tears, respiratory airways, epithelial lining of the alimentary canal, enamel organs, and most significantly in human saliva [51–55]. The physiological role of CA VI has not been established although it has been suggested that it is required for pH homeostasis of the mouth [56]. Maintenance of proper pH levels in saliva are necessary to protect against enamel erosions and acid neutralization in dental biofilms caused by bacteria [57, 58]. As a result it is suggested that CA VI plays a key role in these pathways. Interestingly, CA VI has also shown to be associated with taste and inhibition of CA VI has been shown to cause irregularities in taste perception or sometimes loss of taste completely [59]. This effect however is restored with exposure to high levels of zinc [60].

The membrane-associated CAs include the transmembrane isoforms: CA IX, XII, and XIV, and GPI-anchored isoform CA IV. CA IV is expressed both in the kidneys and lungs [61] and similarly to CA II, CA IV can interact with the same aforementioned transporters that span the renal cell surface [62]. It has therefore been established that the presence of CA IV in the kidney is necessary for bicarbonate resorption and normal kidney function [30]. Interestingly, mutant forms of CA IV have been shown to be associated with an autosomal dominant form of retinitis pigmentosa despite intrinsic levels of wild-type CA IV not being observed in ocular tissue [63].

Both CA IX and XII are often regarded as the tumor-associated CAs [64]. CA IX however has garnered the majority of the attention due to its intrinsically low level of expression in normal tissues [65], in combination with being a key modulator of tumor growth and survival. Specifically, CA IX acts as a mediator of tumorigenesis, pH control, tumor cell proliferation and migration, and cell adhesion [66–70]. CA IX has been shown to be regulated by tumor hypoxia and has not only been established as prognostic indicator for a variety of cancers but also as a generic anticancer target [71–73]. Similarly, CA XII expression has been observed to be upregulated in multiple tumor tissues but it has not been established as a prognostic marker [74–77]. Unlike CA IX, CA XII also shows a wider range of expression in normal tissue including the kidney, lung, prostate, ovaries, uterine endometrium, breast, and basolateral membrane of gut epithelium [64, 78–80]. Furthermore, it has been postulated that CA XII is important for normal kidney function [81].

CA XIV displays high sequence similarity with CA XII and has been shown to be expressed in most parts of the brain, colon, small intestine, urinary bladder, kidney, and retina [82, 83]. Interestingly, immunohistochemical analysis indicates that there is a strong correlation between CA XIV and CA IV expression suggesting there is functional overlap between the enzymes [84]. CA XIV has been shown to directly interact with membrane-transporters and has been observed to be important for pH balance in muscle and erythrocytes in response to chronic hypoxia. Furthermore, CA XIV activity is shown to be important in terms of hyperactivity of the heart and pH regulation in the retina [85–87].

## 2. Methods

A multiple sequence alignment of all the human *α*-CAs was performed in ClustalW2 [98, 99] and used to generate a cladogram that illustrated the evolutionary relationship between the isoforms. The primary sequence identity (%) and number of conserved residues (among the catalytically active isoforms) were calculated in ClustalW2 [98, 99] using the same sequence alignment information. The coordinate files for different CA II inhibitor-complexes were obtained from the Protein Data Bank (PDB) (http://www.wwpdb.org/) to compare the region in which these inhibitors bind in CA II's active site. One file was selected as a reference for the alignment to the other coordinate files in the molecular graphics program* Coot* [100]. A surface rendition of CA II in complex with each of the inhibitors was generated in* Pymol* [101]. The hydrophobicity scores for the residues constituting the hydrophobic cleft were calculated based on the Kyte-Doolittle hydropathy plot [102]. All figures were generated in* Pymol* [101].

## 3. Results and Discussion

### 3.1. Enzyme Inhibition

**Figure 4:**
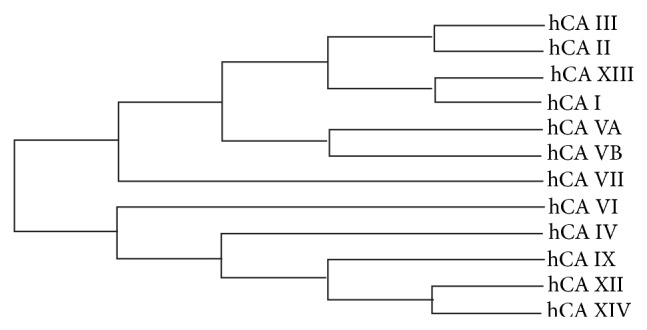
Cladogram of the human *α*-CAs.

The *α*-CAs are very closely related (Figure 4) as per a >30% primary sequence identity amongst them (Table 2). It is this similarity that leads to complications when designing CAIs that are isoform selective as a majority of the sequence identity translates to residues located in the CA active site.  Table 3 shows the number of conserved residues among the different isoforms for residues in the active site and surrounding areas. For example, the 60.5% primary sequence identity that exists between CA I and II (Table 2), in combination with both enzymes being expressed in RBCs, makes CA I a potential off-target isoform when targeting CA II for inhibition [103, 104]. Likewise, when designing selective inhibitors against CA IX, unwanted targeting of CA I and II (with 33.1 and 34.2% identity, resp.) can occur leading to an induced susceptibility to side-effects [9, 105]. The same is true when considering CA VI inhibition where CA II acts as an off-target isoform (33.5% identical) [9].

**Figure 5:**
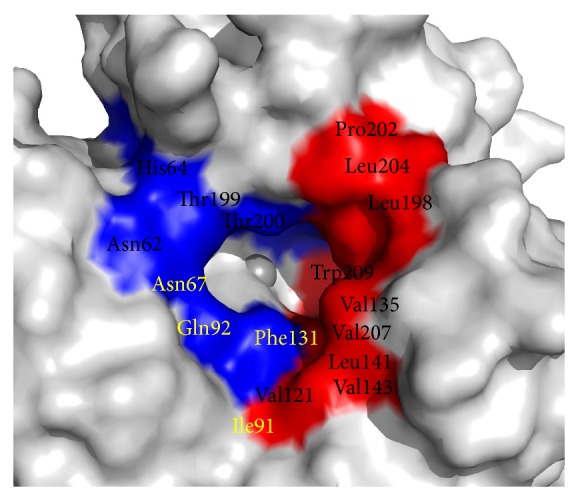
Solvent accessible residues in and around CA II active site. Hydrophilic cleft (blue) and hydrophobic cleft (red). Residues in yellow indicate residues of the “selective pocket.”

Therefore, to design highly selective CAIs requires the exploitation of subtle active site differences; predominantly residues found in the hydrophilic and hydrophobic pockets [22] (Figure 4). Comparative analysis of structures of ligand bound CA molecules shows that exploitable residues that contribute to ligand stabilization include residues N67, I91 and F131 (Figure 5), which are also highly variable between isoforms (Table 4). In addition, Q92, though conserved, has also shown to be important in inhibitor binding. Furthermore, structural interpretation of ligands bound to CA II show that inhibitors can extend out of the active site and form extensive and unique contacts with residues of either the hydrophilic or hydrophobic pocket.

### 3.2. Classical Inhibitors

**Figure 6:**
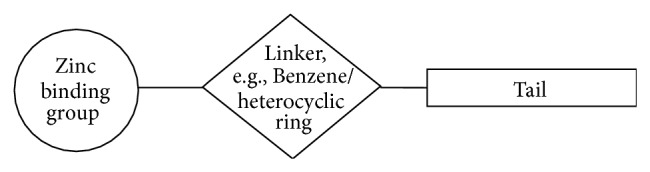
Schematic of the components of a classical CA inhibitor.

Both the catalytic and inhibition mechanism of the *α*-CAs have been studied for several decades and have aided in designing potent isoform specific inhibitors that are important in a wide range of clinical applications (Table 1). This includes CAIs used such as antiglaucoma, antiepileptic, and antiobesity agents, as well as diagnostic tools [41]. A schematic of the basic components of a typical CAI is illustrated in Figure 6. It consists of a zinc-binding group (ZBG), a linker region (heterocyclic or benzene ring) and a variable “tail” region.

As discussed previously CAIs that bind directly to the Zn(II) ion can be divided into two groups based on how they coordinate to the metal center. Those that form trigonal-bipyramidal adducts through way of binding directly to the zinc-bound hydroxyl/water (e.g., cyanates and formates) [9, 16, 22], and those that form tetrahedral adducts and interact directly to the catalytic zinc (e.g., sulfonamides and bisulfites) (Figure 3) [9, 16, 22].

The classical CAIs: the metal-chelating anions and the sulfonamides and their isoesters (sulfamides/sulfamates) are the most studied of the CAIs [22]. However, “nonclassical” CAIs that do not bind directly to the Zn(II) ion also exist. This includes compounds such as coumarins and nitrates [106].

### 3.3. Metal-Chelating Anions

The inorganic anions (e.g., Br^−^) are weaker inhibitors than the sulfonamides and have inhibition constants (*K*_*i*_'s) in the millimolar to submillimolar range [9]. However, for certain isoforms some anions show binding affinities in the low micromolar range (e.g., azide, cyanate, and trithiocarbonate) [88, 107–109]. Unlike the sulfonamides the anions may bind to the metal ion in three different coordination geometries: trigonal-bipyramidal geometry, tetrahedral geometry, or in a distorted tetrahedral geometry. The ability to bind in multigeometries is due primarily to the ligand's structural features. For example, hydrogen sulfide's (HS^−^) ability to act as an H-bond donor to Thr199 allows it to displace the hydroxyl bound zinc and maintain a tetrahedral coordination [9]. On the other hand, unprotonated ligands such as azide (N_3_^−^) and bromide (Br^−^) adopt either the trigonal bipyramidal geometry or distorted tetrahedral geometry [9, 16, 22]. These inhibitors lack the ability to form H-bonds with the O*γ* of Thr199 and so the geometry about the zinc sphere is distorted from the regular tetrahedral geometry [110, 111]. Formate and thiocyanate anions bind as a bipyramidal adduct shifting the zinc bound solvent [12, 112]. Other anions like the nitrates are not coordinated to the metal ion and instead are located in close proximity to it [9, 106].

### 3.4. Sulfonamide-Based CAIs

The sulfonamide*-*based compounds and their isoesters (sulfamides/sulfamates) are by far the most widely represented and clinically used CAIs. This class consists of several compounds, many of which have adapted long-term clinical applications [22]. Brinzolamide, dorzolamide, acetazolamide, methazolamide, and zonisamide have been used as antiglaucoma agents, diuretics, and antiepileptics [9]. Sulfonamides and their bioesters are potent inhibitors with* K*_*i*_'s in the nanomolar range and bind in deprotonated forms to the Zn(II) ion displacing the zinc-bound hydroxyl/water while maintaining a tetrahedral coordination about the active site (Figure 3(c)) [113]. X-ray crystallographic structures of CA I, CA II, and CA IV in complex with these sulfonamide inhibitors are available in the PDB and in all complexes the deprotonated sulfonamide group is coordinated to the Zn(II) ion, while the O*γ* atom of Thr199 makes a hydrogen bond with the sulfonamide's NH moiety. Thr199 also forms a second hydrogen bond to the carboxylate group of Glu106 [16]. Depending on the nature of the R-group, additional interactions with hydrophobic and/or hydrophilic residues in the region of the active site also influence inhibitor binding. However, it is the combination of the negative charge of the monoprotonated sulfonamide group with the positively charged zinc coupled with the ability of Thr199 to form two strong H-bonds that lends the sulfonamides their unique potency for CA inhibition [9].

### 3.5. Nonclassical CAIs

Aside from the classical metal chelating anion and sulfonamide-based inhibitors, which currently represent the majority of CAIs, other potent inhibitors exist. These include thiocarbonates, phenols [114, 115], coumarins [116, 117], polyamines [118], carbohydrate-based sulfonamide derivatives [119–121], and steroid sulfatases [122]. In addition peptidomimetic and monoclonal antibody CAIs have also been utilized [123–125].

The thiocarbamates are anion based chemotypes that exhibit monodentate coordination by way of one sulfur atom binding to the Zn(II) ion in the CA active site. This interaction is coupled with a hydrogen bond observed between an adjacent sulfur molecule reacting with Thr199 [126]. Several compounds currently exist of this chemotype that display nanomolar affinity for CA II and other isoforms. Structural data show that these compounds make unique contacts with several amino acids in the enzymes hydrophilic and hydrophobic binding pockets that can be exploited for design of isoform specific CAIs [127]. Other interesting “nonclassical” CAIs, the phenols, show an alternative mode of binding that is different from both classical sulfonamides and most anions (Figure 8(d)). These compounds anchor directly to the zinc-bound water molecule/hydroxyl rather than the Zn(II) ion itself [114]. However these compounds exhibit a reduction in potency typically in the millimolar range, but there is still a large interest to develop these compounds into potent isoform selective CAIs as they are derived from natural products [128].

Other forms of nonclassical CAIs are the coumarins, which have been both engineered synthetically and isolated as natural products. These compounds vary in regards to isoform inhibition and selectivity [116, 117]. Coumarins, unlike classical CAIs, exhibit “prodrug” characteristics where, prior to binding to the active site, they are hydrolyzed by the esterase activity exhibited by CA that further induces binding at the entrance of the enzymes active site (Figure 8(c)) [116, 117]. This mechanism-based binding event of coumarins suggests that these compounds have potential use in CA isoform selectivity [129–134]. Based off of these observations, sulfur-based derivatives of this chemotype have been formulated and labeled as the “sulfocoumarins” [135]. These compounds also exhibit the same mechanism-based mode of CA binding but show increased affinity via the added sulfur moiety, which forms direct interactions with the catalytic zinc [135].

Polyamines, which belong to an alkaloid structural class, have also shown utility as CAIs [115, 118]. Several polyamine derivatives that have been isolated display high levels of CA isoform selectivity with potencies ranging from millimolar to low nanomolar levels [118]. Unlike the aforementioned CAIs, polyamines exhibit a mode of binding reliant on hydrogen bond formation throughout the active site cavity. Specifically, they anchor to the zinc-bound water/hydroxide (similar to phenols) with the terminal amine interacting with residues in positions 200 and 201 [118]. Most likely this attribute contributes to isoform selectivity of various polyamine CAIs and can thus be further developed to engineer more specific and potent CAIs of this class.

Several glycosyl primary sulfonamides and glycoconjugate sulfamates have been recognized as CAIs [120, 121]. These compounds are typically modifications of classical sulfonamide CAIs that usually have an aromatic-ring branched to the primary sulfonamide group (Figure 6). Instead these compounds replace the aromatic attachments of primary sulfonamides with mono- or disaccharide moieties [119–121]. Interestingly, the addition of a specific sugar moiety induces variable isoform selectivity ranging from micromolar to low nanomolar levels between CAs. More notably, these compounds have found use in inhibiting tumor associated isoforms IX and XII [119–121]. Not only do these compounds exhibit high affinity for CA IX/XII but the bulky sugar moieties cause a reduction in membrane permeability allowing for selective targeting of the extracellular facing catalytic domain of both tumor associated isoforms thus acting as location specific CAIs [119–121].

Similar to adding bulky-carbohydrate moieties to sulfonamides, steroid sulfatase inhibitors, which have been designed based on previously seen antimitotic inhibitors [136, 137] are able to take advantage of the variable residues in the hydrophobic pocket of specific CAs via van Der Waals contacts of the steroidal backbone [136–138]. The same trend was seen in energy calculations from molecular docking studies of such compounds with CA IX [137]. These particular compounds are also useful in locating specific targeting of extracellular CAs due to their reduced membrane permeability [136, 137].

In addition to the development of small-molecule inhibitors of CAs, there are several biologics used for CA inhibition. Utilization of monoclonal antibodies, such as M75 and G250, to recognize the proteoglycan-like (PG) domain (the N-terminal extension unique to this isoform) of CA IX have shown effectiveness in disrupting the ability of the enzymes function in regulating tumor cell adhesion and motility [139, 140]. More recently, the monoclonal antibody 6A10 has been developed to mediate CA XII activity also acting as a potential anticancer therapeutic [124, 125]. This becomes promising as such monoclonal antibodies exhibit high affinity to their target and can thus be used to distinguish between isoforms [124, 125]. More recently, peptide based inhibitors for CA IX have also been discovered utilizing a phage-display library [123]. However the benefits of these types of ligands are still unclear. Although there is postulation that the specific binding region of such peptides can be further exploited for the development of a biologic drug that is isoform selective [123].

### 3.6. Preferential Binding

**Figure 7:**
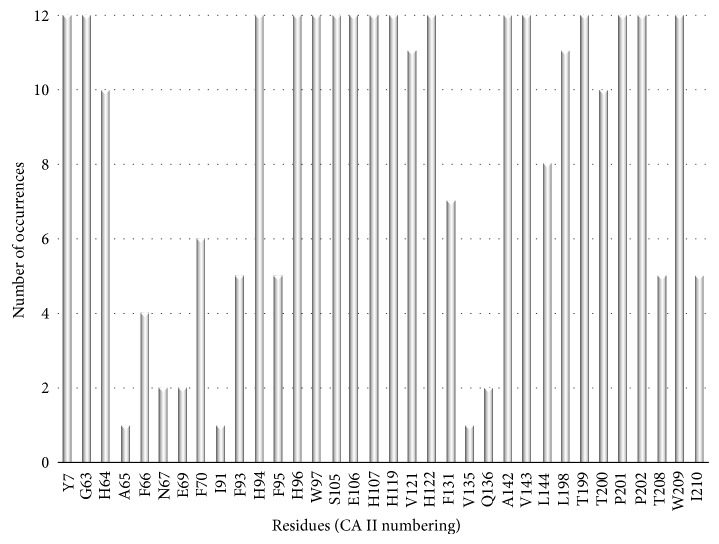
Bar graph of active site residues in the catalytic CA isozymes (CA II numbering).

As we have seen the major hurdle in developing isoform selective CAIs is to design inhibitors that can distinguish between the similarities of the *α*-CA active site architecture. This would require the CAI to have limited interactions with conserved regions of the active site such as the three histidine residues coordinating the Zn(II) ion seen in all 12 catalytically active isoforms, residues that have shown to contribute to inhibitor binding such as Thr199 and Glu106 in CA II, and most of the residues that constitute both the hydrophobic and hydrophilic cleft as they are conserved (Figure 7).

**Figure 8:**
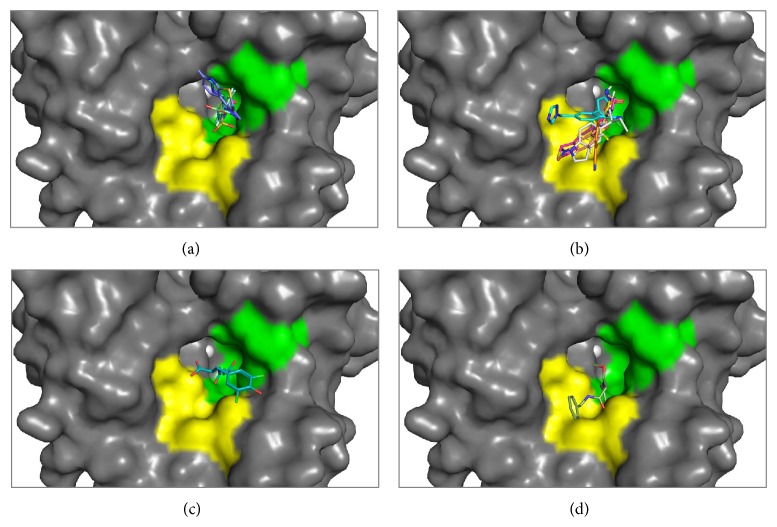
CA inhibitor: (a) several inhibitors binding in the conserved region (green) of CA II's active site. These inhibitors are buried in the active site and are stabilized predominantly by hydrophobic residues (b). Several inhibitors occupying the “selective pocket” (yellow) of CA II. The tails of these inhibitors are extending out of the active site. (c) Coumarin binding on the perimeter of the active site. (d) Phenol binding in the proximity of the active site.

Human CA II is the most well studied and characterized of the CA isoforms [141]. Over 400 X-ray crystallographic structures of CA II (both wild-type and variants) exist in the PDB with over 150 submissions containing CA II inhibitors [106]. Using the CA II active site as a reference it can be observed that the majority of inhibitors are buried deep in the enzymes active site (Figure 8(a)) and are restricted to the highly conserved region, which can be termed the “*conserved pocket*” (green shaded region, Figure 8(a)). Most of these inhibitors are sulfonamides (with short organic scaffolds) and so maintain the tetrahedral coordination about the zinc sphere while the variable “tails” of these inhibitors interact mainly with residues making up the hydrophobic and hydrophilic clefts. Furthermore, these variable “tail” regions are observed to be stabilized by H-bonds and hydrophobic interactions with Thr199, Thr200, Val121, Val143, and Leu198.

Despite the structural similarities observed between the CA isoforms, amino acid differences exist in specific regions of the active site. This region is defined as the “*selective pocket*” [106] (yellow shaded region, Figure 8(b)) and lies towards the edge of the active site relative to the catalytic zinc. Those inhibitors that are restricted to the* conserved pocket* are unable to form interactions with residues residing in the* selective pocket* due to the compact nature of their chemical scaffolds. Simply, the tails of these inhibitors are too short to interact with the residues that constitute the* selective pocket* and therefore cannot establish extensive contacts that can contribute to isoform selective inhibition. Residue positions 67, 91, and 131 establish this region termed the* selective pocket* (Table 3). Gln92, though conserved in all the isoforms, is also instrumental in contributing to inhibitor binding along with these select residues.

In addition to exploiting residues in the* selective pocket* between isoforms, selective CAIs can be designed based on overall hydrophobicity of the active site cleft. For example, CA II and CA IX display the most hydrophobic (hydrophobicity sores of ~26 and ~23, resp.) active site implying that designing CAIs with long flexible tails of a more hydrophobic nature may be beneficial to induce desired selective binding (Table 4). Notably, this attribute of the CA IX active-site coupled with its extracellular location provides an avenue to (1) design more hydrophobic CAIs that favor CA IX binding over other extracellular CAs and (2) engineer more bulky CAIs such that membrane permeability becomes poor thus eliminating the potential for CA II inhibition.

In order to design new isoform specific inhibitors that circumvent off-target CA inhibition, the structural dissimilarities that exist between the isoforms, particularly in the* selective pocket*, can be exploited. In addition, taking advantage of the global hydrophobic nature of the CA II or CA IX active site cleft provides a method to selective CAI design. It is already known that the sulfonamides are the most potent CAIs and this knowledge has been used to develop what is known as the “tail approach” to aid in the development of new inhibitors [142, 143]. This approach involves the appending of variable “tails” to the scaffolds of aromatic/heterocyclic sulfonamides to elongate the molecule. This allows the inhibitor to interact with amino acids from the middle to the edge of the active site relative to the catalytic zinc, which ultimately vary between different isoforms [106]. Small molecules such as phenols (Figure 8(c)) and coumarin (Figure 8(d)) also exhibit this same property by directly interacting with residues of the* selective pocket*.

## 4. Conclusions

A comparison of the conserved and nonconserved regions in the CA catalytic-site between isoforms revealed areas that can be exploited for rational design of selective CAIs. Specifically, highly variable areas amongst active site residues occur outwardly relative to the catalytic zinc in what has been defined as the* selective pocket*. Sequence alignments show that residues in positions 67, 91, and 131 vary between isoforms and structural analysis of CA II in complex with various inhibitors, show that “tails” of inhibitors make extensive contacts with these residues (Figures 5 and 8). Residues at position 91 seem to have the highest variability, in terms of specific residues type and between amino acid properties (i.e., hydrophilicity/hydrophobicity) between isoforms (Table 3). Interestingly, it is observed that CA II and IX exhibit the most hydrophobic catalytic domain and are the only isoforms (with exception to CA I and XIV) that contain hydrophobic residues at this position as well (Leu91 in CA IX). Position 91 can be termed a “hot-spot” for the design of isoform specific inhibitors, such that it contains both high variations between physical properties of amino acid, but (in the case of CA II and IX) there is also observable variation specific side-chain associated with the residues in this position. This attributes position 91 as being a key area that can be exploited by specific chemotypes and thus provides an alternative path for the design of selective CAIs. Overall, it is observed in Figure 8(b) that the residues farthest from the catalytic domain (relative to the zinc) remain the least conserved. This provides an exceptional advantage to the rational design of isoform specific inhibitors in that these variable regions can also be exploited by specific chemotypes. This notion is analogous to the idea of utilizing sulfonamide inhibitors with variable “tail” regions for isoform selective inhibitor development however in this study we have presented a more guided approach to this method of CAI design [106].

In summary our observations provide a template to exploit the variable regions of the catalytic domains of different CA isoforms. These guidelines can be utilized for the development of classical and nonclassical CAIs to overcome the potential of off-target CA inhibition and further lead to the development of more selective CAIs that can be employed in the clinic.

## Supplementary Material

Surface rendition of carbonic anhydrase II showing how various inhibitors bind in and around the active site cleft. The “conserved region” (green) and “selective pocket” indicate regions of preferred binding by various carbonic anhydrase inhibitors.

## Figures and Tables

**Table 1:** Distribution, associated diseases, catalytic efficiency, and structural characterization of CAs.

Isoform	Localization		*K*_cat_ (s^−1^)	*K*_cat_/*K*_*M*_ (M^−1^ s^−1^)	p*I*	Oligomeric state	Number of PDB entries	References
	Organ/tissue	Subcellular						
		Associated disease						
I	RBCs, GI tract, and eye	Cytosol	2.0 × 10^5^	5.0 × 10^7^	6.6	Monomer	19	[2, 3, 9, 88, 89]
		Hemolytic anemia						

II	RBCs, kidney, osteoclasts, eye, GI tract, lung, brain, and testis	Cytosol	1.4 × 10^6^	1.5 × 10^8^	6.9	Monomer	454	[2, 3, 3, 9, 88–90]
		Glaucoma, epilepsy, edema, altitude sickness						

III	Adipocytes, skeletal muscle	Cytosol	1.0 × 10^4^	3.0 × 10^5^	7.0	Monomer	6	[2, 3, 9, 88, 89]
		Oxidative stress						

IV	Lung, kidney, brain, eye, RBCs, and colon	Membrane-bound	1.1 × 10^6^	5.1 × 10^7^	6.4	Monomer	4	[3, 9]
		Retinitis pigmentosa, stroke, glaucoma						

VA	Liver	Mitochondria	2.9 × 10^6^	2.9 × 10^7^	7.2	Monomer	1^*^	[4, 91]
		Obesity, insulin resistance						

VB	Kidney, GI tract, spinal cord, heart and skeletal muscle, and pancreas	Mitochondria	9.5 × 10^5^	9.8 × 10^7^	7.7	Monomer	N/A	[4, 91]
		Obesity, insulin resistance						

VI	Salivary and mammary glands	Secreted	3.4 × 10^5^	4.9 × 10^7^	6.5	Dimer	1	[5, 9, 92, 93]
		Dental caries						

VII	Liver, colon, skeletal muscle, and brain	Cytosol	9.5 × 10^5^	8.3 × 10^7^	6.9	Monomer	2	[6, 9, 94]
		Epilepsy						

IX	GI mucosa, tumors	Transmembrane	3.8 × 10^5^	5.5 × 10^7^	5.5	Dimer	2	[3, 7, 9, 88, 89, 95]
		Cancer						

XII	Eye, tumors, reproductive epithelia, intestines, and kidney	Transmembrane	4.2 × 10^5^	3.5 × 10^7^	5.8	Dimer	5	[3, 8, 96]
		Cancer, glaucoma						

XIII	Kidney, thymus, submandibular glands, small intestine, and reproductive organs	Cytosol sterility	1.5 × 10^5^	1.1 × 10^7^	6.5	Monomer	6	[5, 44]

XIV	Eye, brain, kidney, liver, bladder, and spinal cord	Transmembrane	3.1 × 10^5^	3.9 × 10^7^	5.5	Monomer	1	[9, 97]
		Retinopathy, epilepsy						

^*^murine.

**Table 2:** Primary sequence identity (%) (lower left) and number of conserved residues (upper right) among catalytic CAs.

	I	II	III	IV	VA	VB	VI	VII	IX	XII	XIII	XIV
I	—	158	141	78	126	128	82	132	83	91	154	85
II	60.5	—	152	88	133	138	90	147	85	89	157	96
III	54.2	58.5	—	82	120	117	87	130	80	86	151	90
IV	30.0	33.5	31.2	—	89	93	97	90	84	91	84	62
VA	48.1	50.8	45.4	23.6	—	184	93	131	83	84	124	88
VB	46.9	51.9	43.5	23.1	58.7	—	82	134	89	79	131	88
VI	31.9	33.5	32.3	27.0	27.9	24.4	—	93	107	104	90	106
VII	50.8	56.2	49.6	31.8	48.5	49.2	34.9	—	95	103	139	97
IX	33.1	34.2	31.1	27.2	31.9	32.7	38.9	37.0	—	101	90	113
XII	35.8	34.2	32.3	28.1	31.6	29.7	38.0	38.0	38.9	—	91	123
XIII	59.2	59.6	57.7	28.2	46.2	47.7	33.2	52.7	35.0	34.7	—	98
XIV	34.2	35.8	34.2	29.0	31.9	29.0	35.8	36.0	44.4	46.0	37.4	—

**Table 3:** Active site residues of catalytic CAs (*CA II numbering*).

Residues	Isozyme										
	I	III	IV	VA	VB	VI	VII	IX	XII	XIII	XIV
Y7	Y	Y	Y	W	Y	Y	Y	Y	Y	Y	Y
N62	N	N	N	N	N	N	N	N	N	N	N
N67^*^	H	R	M	Q	L	Q	Q	Q	K	N	Q
I91^*^	F	R	K	K	K	Q	K	L	T	R	A
Q92	Q	Q	Q	Q	Q	Q	Q	Q	Q	Q	Q
H94	H	H	H	H	H	H	H	H	H	H	H
H96	H	H	H	H	H	H	H	H	H	H	H
H119	H	H	H	H	H	H	H	H	H	H	H
V121	A	V	V	V	V	V	V	V	V	V	V
F131^*^	L	F	V	Y	F	Y	F	V	A	F	L
V135	A	L	Q	V	A	Q	A	L	S	A	A
V143	V	V	V	V	V	V	V	V	V	V	V
L198	L	F	L	L	L	L	L	L	L	L	L
T199	T	T	T	T	T	T	T	T	T	T	T
T200	T	T	T	T	T	T	T	T	T	V	T
P202	P	T	P	P	P	P	P	P	P	P	P
W209	W	W	W	W	W	W	W	W	W	W	W

^*^residues making up the selective pocket.

**Table 4:** Hydrophobicity of CA active sites (*CA II numbering*).

Residues	Isozyme										
	I^1^	II^2^	III^3^	IV^4^	V^5^^*^	VI^6^	VII^7^	IX^8^	XII^9^	XIII^10^	XIV^11^
I91	F	I	R	K	K	Q	K	L	T	R	A
V121	A	V	V	V	V	V	V	V	V	V	V
V135	A	V	L	Q	S	Q	A	L	S	A	A
V141	L	L	L	I	L	L	L	L	L	L	L
V143	V	V	V	V	V	V	V	V	V	V	V
L198	L	L	F	L	L	L	L	L	L	L	L
P202	P	P	T	P	P	P	P	P	P	P	P
L204	Y	L	E	D	A	T	S	A	N	L	Y
W209	W	W	W	W	W	W	W	W	W	W	W

Total hydrophobicity	**14**	**26**	**8**	**4**	**11**	**7**	**11**	**23**	**9**	**15**	**16**

^1^2FOY; ^2^3KS3; ^3^3UYN; ^4^1ZNC; ^5^1DMX ^*^murine; ^6^3FE4; ^7^3MDZ; ^8^3IAI; ^9^1JC2; ^10^3DAZ; ^11^4LU3.
