# Supplementary material for: Probing the Surface of Human Carbonic Anhydrase for Clues towards the Design of Isoform Specific Inhibitors
Source: Biomed Res Int. 2015 Feb 24;2015:453543. doi: 10.1155/2015/453543 (PMC4355338; doi:10.1155/2015/453543)
Supplement: Supplementary file 1 — Surface rendition of carbonic anhydrase II showing how various inhibitors bind in and around the active site cleft. The “conserved region” (green) and “selective pocket” indicate regions of preferred binding by various carbonic anhydrase inhibitors. [file 453543.f1.pdf]

Supplementary Materials

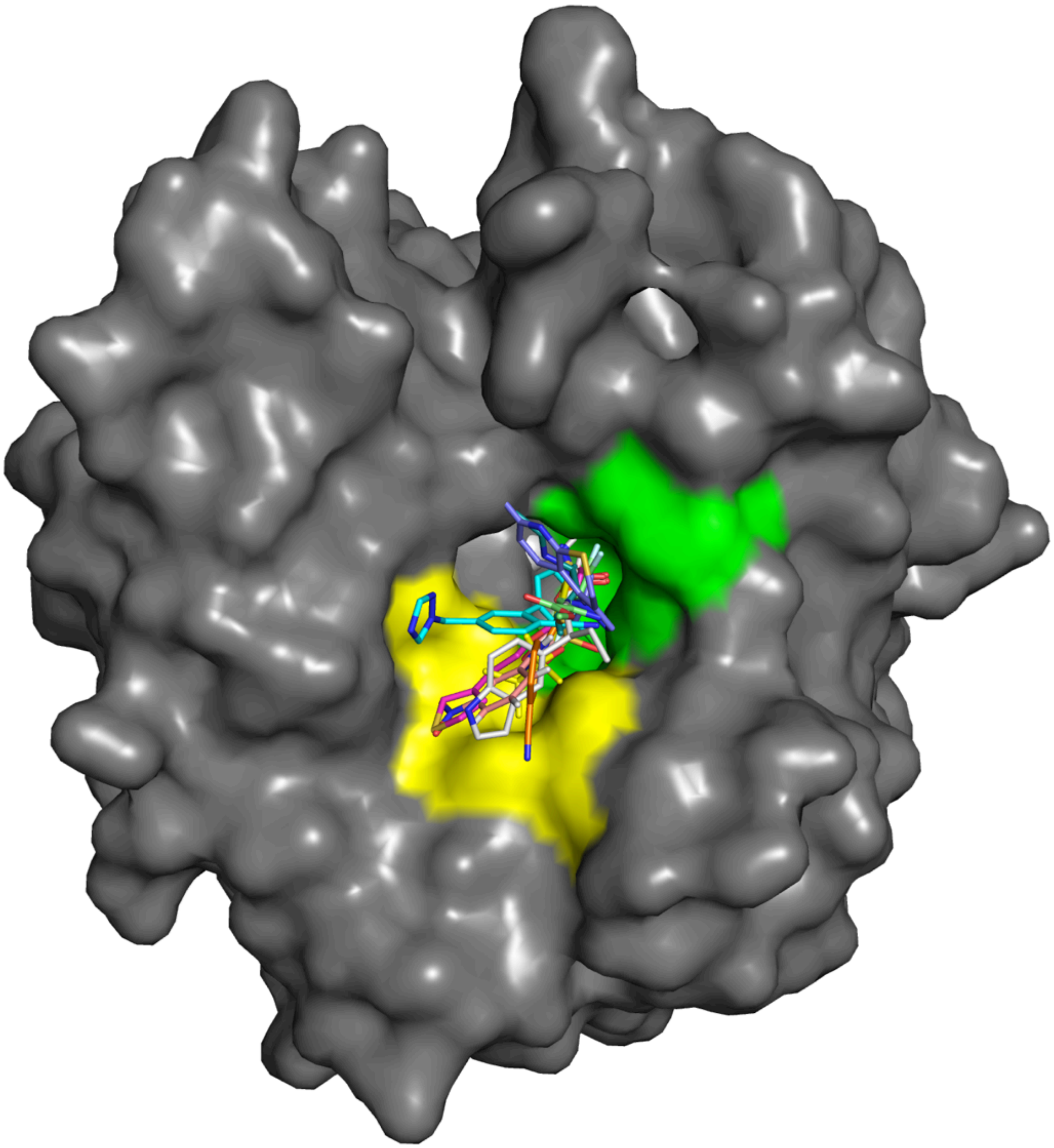

Binding of various inhibitors in the “conserved region” (green) or “selective pocket” (yellow) of the carbonic anhydrase II active site.
